# Intramuscular Electrical Stimulation for the Treatment of Trigger Points in Patients with Chronic Migraine: A Protocol for a Pilot Study Using a Single-Case Experimental Design

**DOI:** 10.3390/medicina59081380

**Published:** 2023-07-28

**Authors:** Thomas Perreault, Lars Arendt-Nielson, César Fernández-de-las-Peñas, Jan Dommerholt, Pablo Herrero, Ryan Hubbard

**Affiliations:** 1 Department of Physical Therapy, Wentworth Douglass Hospital, Dover, NH 03820, USA; rshubbard@mgb.org; 2 Center for Neuroplasticity and Pain, SMI, School of Medicine, Aalborg University, DK-9220 Aalborg, Denmark; lan@hst.aau.dk (L.A.-N.); cesar.fernandez@urjc.es (C.F.-d.-l.-P.); 3 Department of Gastroenterology & Hepatology, Mech-Sense, Clinical Institute, Aalborg University Hospital, DK-9000 Aalborg, Denmark; 4 Steno Diabetes Center North Denmark, Clinical Institute, Aalborg University Hospital, DK-9000 Aalborg, Denmark; 5 Department of Physical Therapy, Occupational Therapy, Rehabilitation and Physical Medicine, Universidad Rey Juan Carlos, 28922 Madrid, Spain; 6 Cátedra Institucional en Docencia, Clínica e Investigación en Fisioterapia-Terapia Manual, Punción Seca y Ejercicio Terapéutico, Universidad Rey Juan Carlos, 28922 Madrid, Spain; 7 Myopain Seminars, Bethesda, MD 20814, USA; jan@myopainseminars.com; 8 Department of Physical Therapy and Rehabilitation Science, School of Medicine, University of Maryland, Baltimore, MD 21201, USA; 9 IIS Aragon, Department of Physiatry and Nursing, Faculty of Health Sciences, University of Zaragoza, 50009 Zaragoza, Spain; pherrero@unizar.es

**Keywords:** intramuscular, electrical stimulation, chronic migraine, trigger points

## Abstract

*Background and Objectives:* Trigger points (TrPs) are prevalent in patients with migraine headaches. Needling interventions targeting TrPs in migraine patients may reduce the intensity and frequency of headaches, yet systematic reviews reveal a lack of robust evidence. Intramuscular electrical stimulation (IMES) is a modality that delivers electrical current into muscles and TrPs, with recent studies suggesting it may amplify the therapeutic effects of dry needling peripherally and centrally. This could be advantageous for patients with migraine and symptomatic TrPs. *Materials and Methods:* This study will implement a multiple baseline single-case experimental design (SCED). In a clinical setting, a SCED study lends itself to conducting research with only a few patients that each serve as their own controls. In this SCED study, four participants with chronic migraine will be enrolled in a non-concurrent manner and randomized to one of four baseline measurement periods (4, 5, 6 or 7 weeks), leading to four potentially different start dates for each participant in the intervention phase. During the intervention phase, patients will receive five sessions of dry needling with IMES, one session per week for five weeks. The primary outcome measure will be headache frequency, i.e., the reduction in the number of headache days over a one-month period using electronic headache diary data from the Migraine Buddy smartphone application. Secondary outcome measures will be changes in mean migraine pain intensity using a numeric pain rating scale (NPRS), migraine disability using the Migraine Disability Assessment Test (MIDAS), the Headache Impact Test (HIT-6), and changes in selected cervical musculoskeletal impairments including pressure pain thresholds (PPTs) over TrPs, the craniocervical flexion test (CCFT), and cervical active range of motion (AROM). Primary and secondary outcome measures will be analyzed separately using both visual and statistical analyses. *Results*: Actively recruiting participants. This project was approved by the Mass General Brigham Institutional Review Board (protocol #2023P000931) and is registered with ClinicalTrials.gov (NCT05893914). *Conclusions*: This study will seek to determine the effects of a five-week intervention period of IMES to TrPs in the posterior cervical muscles of subjects with chronic migraine.

## 1. Introduction

Migraine is a recurrent headache disorder characterized by attacks lasting 4–72 h with a range of associated symptoms and degrees of severity [1]. It is a highly prevalent disorder and a leading cause of disability worldwide [2]. Migraine has an estimated global prevalence of 14–15% [3], and in the United States, the three-month prevalence rate is reported to range from 16.6% to 22.7%, according to data from large-scale surveillance studies [4]. There are two major migraine types, migraine with or without aura. In addition, migraine can be further defined depending on the number of headache days experienced per month, with chronic migraine sufferers experiencing fifteen or more days with migraine attacks, and often with higher pain intensity and overall headache impact [5]. Global prevalence estimates of chronic migraine range between 1.4 and 2.2% [6]. The pathophysiology of migraine is complex and includes sensitization of the thalamus and brainstem, and nociceptive inputs from afferent nerves that converge onto the trigemino-cervical complex (TCC) [7]. In many cases, neck pain accompanies migraine, with a recent study reporting a frequency of 77% and neck pain being twice as likely in patients with chronic compared to episodic migraine [8]. Although not all neck pain in migraine is associated with specific cervical musculoskeletal impairments [9], it is suggested that persistent nociceptive input from certain impairments in the cervical area may lead to further sensitization of the TCC, contributing to migraine attacks [10,11,12].

Several studies have demonstrated a high prevalence of trigger points (TrPs) in patients with migraine compared to healthy controls [13] and in migraine patients with neck pain compared to migraine without neck pain [14]. Trigger points are painful spots within taut bands of skeletal muscles that produce local and referred pain either spontaneously or upon stimulation [15]. Meng et al. found that TrPs contribute to central sensitization and cause plastic changes within nociceptive pathways [16]. A previous study reported that TrPs are common in the temporal and suboccipital muscles and when stimulated often reproduce a headache pain that is familiar to the patient [17]. A recent study on females with episodic migraine also found TrPs in the head, neck, and shoulder girdle muscles that were associated with widespread pressure hypersensitivity, including pressure sensitivity over articular regions of the cervical spine [18]. Based on the available evidence, TrPs in muscles of the head and neck may contribute to migraine due to nociceptive inputs that project along cervical afferent pathways that converge onto neurons within the trigeminal nucleus caudalis, leading to referred pain in the head [19,20].

Needling interventions targeting TrPs in patients with migraine show favorable reductions in the intensity and frequency of headaches [21,22,23]. Yet, systemic reviews reveal a sparsity of studies, most with small sample sizes, for the treatment of TrPs in migraine [24,25]. Intramuscular electrical stimulation (IMES) is an electrotherapeutic modality that uses needles to deliver an electrical current into muscles and, more specifically, into TrPs [26]. Moreover, IMES is a form of neuromodulation, i.e., it uses electrical stimulation to modulate the activity of peripheral and central neural pathways to treat pain. It is proposed to have mechanistic effects on TrPs by normalizing muscle blood flow, offsetting local ischemia and hypoxia, and normalizing electrical activity at the motor endplate. Supraspinal mechanisms of IMES include improving function of the descending pain modulatory system [27,28]. The use of an electrical current applied to needles may reduce local muscular injury, inflammatory reactions, and post-needling soreness compared to the repeated manual needle stimulation commonly used to treat TrPs [20].

To our knowledge, no studies have investigated the effects of IMES on TrPs in the posterior cervical muscles in patients with chronic migraine. This exploratory study will implement a single-case experimental design (SCED). The use of a SCED study lends itself to conducting research in a clinical setting with few patients that serve as their own controls. Single-case experimental design studies have long been used in the education and psychology professions, but more recently have been implemented in medical and rehabilitation research, including physical therapy [29,30]. In SCED studies, a multiple baseline design across patients is commonly used to demonstrate the stability of a particular condition prior to the staggered introduction of each patient to the intervention phase [31]. Repeated measurements of a target variable, along with other relevant measures, are taken in all phases, and the study power is determined by the number of repeated measures, and not by the number of total participants [32]. Changes observed in the intervention phase are analyzed against the baseline, within and between patients, and displayed through visual and statistical means [29,33]. Importantly, SCED studies are patient-centered, and allow a more personalized care approach in the research process compared to more robust randomized controlled trials [30]. Patient-centered and individualized approaches for the treatment of patients with migraine are recommended [34].

The primary aim of this proposed pilot study using a single-case experimental design is to investigate the effects of five weeks of IMES for the treatment of TrPs on the frequency of headaches in a small sample of patients with chronic migraine. Additionally, the secondary aims of this study will be to evaluate the effects of IMES on mean headache intensity, overall headache impact, disability, and the influence of IMES on various cervical musculoskeletal impairments.

## 2. Methods

### 2.1. Study Design

**Figure 1:**
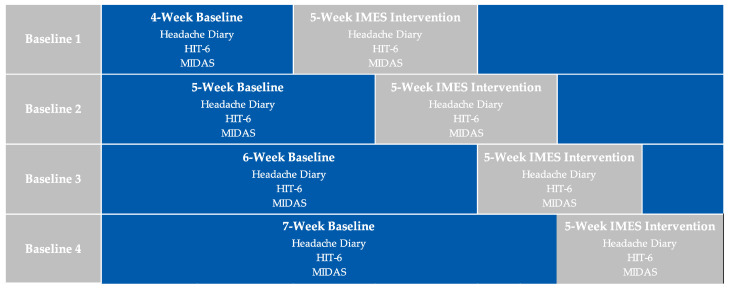
Baseline tiers 1–4, each followed by the intervention phase.

This study protocol and subsequent experiment will follow the Single-Case Reporting Guideline in Behavioral Interventions (SCRIBE) 2016 [35]. This project was approved by the Mass General Brigham Institutional Review Board (protocol #2023P000931) and is registered with ClinicalTrials.gov (NCT05893914). This study will implement a single-case, non-concurrent, multiple-baseline experimental design, using an AB design (A = baseline, B = treatment). To strengthen the internal validity of this experiment, participants will be randomly assigned to one of four baseline tiers, leading to four potentially different start dates for each participant in the intervention phase. The first tier will include 4 weeks (28 days) of baseline measurements (phase A), followed by 5 weeks of treatment and symptom monitoring (phase B); the second tier will include 5 weeks (35 days) of baseline measurements (phase A), followed by 5 weeks of treatment and symptom monitoring (phase B); the third tier will include 6 weeks (42 days) of baseline measurements, followed by 5 weeks of treatment and symptom monitoring (phase B); and the fourth tier will include 7 weeks (49 days) of baseline measurements, followed by 5 weeks for treatment and symptom monitoring (Figure 1). According to the International Headache Society, a 28-day minimum prospective baseline period using a headache diary is recommended to establish the attack frequency in a study of chronic migraine [36]. The length of the baseline and follow-up periods were chosen to ensure a minimum of 4 to 5 assessments per participant per phase, as recommended for the analyses of SCEDs [31]. In total, the duration of the study will be 9–12 weeks depending on the baseline assignment. In the intervention phase (phase B), all participants will receive one intervention session per week, over five weeks.

### 2.2. Study Setting and Participants

This SCED study will recruit four participants with chronic migraine, in a non-concurrent manner. A recent systematic review recommended an average of four participants across SCED studies using a multiple-baseline design [37]. Patients being referred for rehabilitation services at Wentworth Douglass Hospital Outpatient Physical Therapy Department with a diagnosis of migraine will be screened for eligibility. Preliminarily eligible participants will be contacted via phone by the principal investigator, prior to their first scheduled physical therapy visit, to be informed of the study. A standard study description will be used to explain the purpose of the study to participants, including benefits and risks. As part of the screening tool, patients will be asked if they have a needle phobia that would interfere with their ability to participate in study visits. A previous dry needling study reported no difference in pain tolerance between patients with or without a fear of needles [38]. However, if participants answer yes to having a needle phobia in the screening process, they will be excluded.

### 2.3. Participant Selection

#### 2.3.1. Inclusion Criteria

To be eligible, patients must be adults between the ages of 18 and 65 years. Participants will be included if they have at least a 6-month history of headaches consistent with a diagnosis of chronic migraine headache according to International Headache Society (IHS) criteria [1], as determined by a neurologist. That is, headaches occurring on 15 or more days/month for more than three months, which, on at least eight days/month, have the features of a migraine headache. In addition, participants must have active TrPs ipsilateral to the side of headache and reproducing headache symptoms upon stimulation within the distribution of headache in at least 2 of the following posterior cervical muscles: splenius capitis, splenius cervicis, semispinalis capitis/cervicis, cervical multifidi, and obliquus capitis inferior.

#### 2.3.2. Exclusion Criteria

Participants will be excluded if they have other primary or secondary headache diagnoses, including medication overuse headache; cervical radiculopathy; cervical spondylosis or stenosis; previous surgery in the cervical spine region; history of whiplash; pregnancy; a current diagnosis of fibromyalgia; received TrP injection, anesthetic blocks, radiofrequency lesioning, botulinum toxin injections, acupuncture, dry needling, or physical therapy treatment within the past 12 months for the treatment of neck pain or migraine headache.

### 2.4. Participant Evaluation

Before the clinical examination procedures begin, eligible patients based on pre-screening criteria, must sign the informed consent form indicating their willingness to participate in the study. Once signed, all participants will first undergo a comprehensive subjective history followed by various clinical examination tests to determine full eligibility, which will all be completed by the principal investigator who is a licensed Physical Therapist with board certification as an Orthopedic Clinical Specialist with over 15 years of clinical experience. All participants will undergo a manual examination for TrPs in the posterior cervical muscles using diagnostic criteria supported by expert consensus [39]. Through palpation, TrPs will be identified by the presence of a hypersensitive spot in a taut band of skeletal muscle and referred pain or sensation upon stimulation [40]. A recent systematic review reported uncertainty with recommending TrPs as an entity in the clinical examination of patients with migraine, yet TrPs were frequently assessed in the included studies, and the recommendations were limited only by study heterogeneity and inadequate results reporting [41]. Stimulation to TrP areas will be performed to reproduce any symptom experienced by the patient, either partially or completely, whereby the symptom is recognized as a familiar experience by that patient. i.e., in this case, reproduction of migraine attack symptomatology [13,18,39]. In addition, the pressure pain thresholds (PPTs) will be assessed in the location of the TrPs using an analogue algometer (Force Dial FDK 20, Wagner Instruments, Greenwich. Connecticut), which has shown good concurrent validity in patients with migraine [42]. Patients with migraine headache show decreased PPT levels over local areas in the cervical musculature [43], including the suboccipital muscles [44].

To ensure a comprehensive examination of the cervical spine in participants with migraine, cervical range of motion, manual joint testing, and the flexion-rotation test (FRT) will be assessed [41]. In addition, the cranio-cervical flexion test (CCFT) will be used to assess performance and isometric endurance of deep cervical flexor muscles. According to a recent study, the CCFT has shown acceptable discriminative validity in discerning individuals with migraine and asymptomatic controls [45]. Furthermore, a combination of findings on the CCFT, FRT and upper cervical joint palpation, may help to detect the likelihood of cervicogenic headache and aid in excluding patients without clear findings upon TrP assessment [46]. Cervical spine active range of motion (AROM) will be measured using a JAMAR E-Z Read goniometer (Patterson Medical, Warrenville, IL, USA), a plastic universal twelve-inch, double-armed 360° goniometer for cervical rotation, and a Baseline bubble inclinometer (Fabrication Enterprises, White Plains, NY, USA) for all other motions. Both are standard clinical devices that are reported to have good reliability for clinical use [47,48]. Segmental mobility will be tested using a lateral gliding [49] and segmental side-bending maneuvers [50,51] in order to examine patients for hypomobility of the C2–C4 segments. Flexion rotation test (FRT) will be performed bilaterally to assess for pain provocation and impaired mobility of the upper cervical spine at the C1–2 levels [46], which has been reported in patients with migraine [52]. Ligamentous safety tests to assess the alar and transverse ligaments will be assessed including the side-bending stress test [53] and anterior shear test [54,55] in order to screen for cranio-cervical instability. A neurological examination will be performed to screen for any findings that would indicate cervical radiculopathy and will consist of deep tendon reflex testing; dermatome sensation testing; and myotome testing, except for the C1–C4 levels, which are difficult to assess clinically via manual muscle testing [56,57].

### 2.5. Interventions

**Figure 2:**
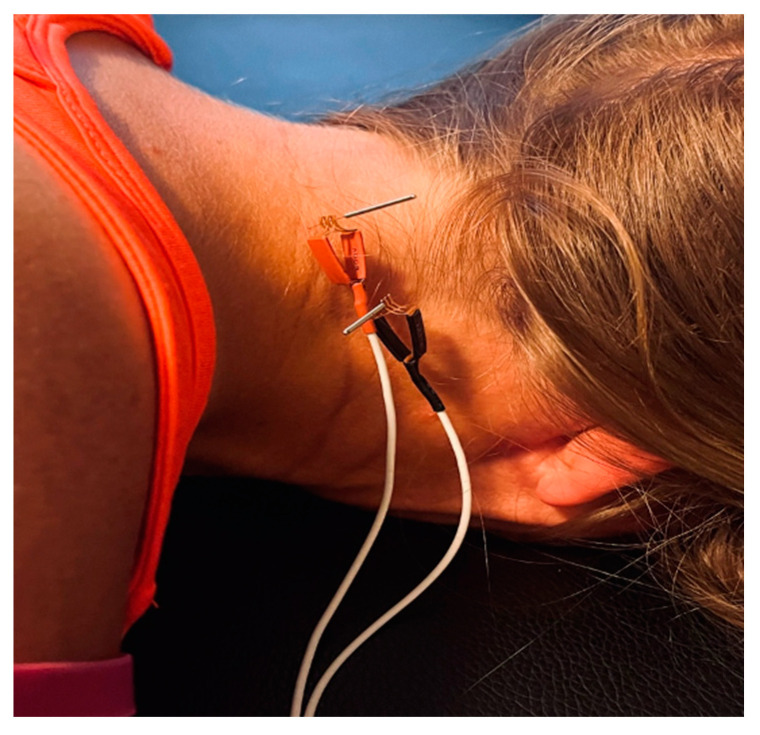
Dry needling with IEMS to posterior cervical muscles.

During the intervention phase, patients will receive five sessions of dry needling with IMES, one session per week for five weeks total during the intervention phase. Dry needling will be delivered by a Physical Therapist (principal investigator) who has over 10 years of clinical dry needling experience and is certified by a course program that provides 80 h of dry needling instruction. Sterilized disposable stainless-steel needles will be used and will include one standard size, 0.30 mm × 50 mm APS needles (Agupunt, Barcelona, Spain). Dry needling will be applied to TrPs in at least two of the following posterior cervical muscles: splenius capitis, splenius cervicis, semispinalis capitis/cervicis, cervical multifidi (from C2–4), and obliquus capitis inferior. These muscles were chosen because they receive innervation from the upper cervical dorsal rami and therefore may influence the TCC. Trigger points will be treated on the side of the headache or bilaterally if they are present. For the posterior cervical muscles, they will be treated as one muscle group with all needling to be performed posterior to the transverse processes and lateral to the spinous processes of C2 to C6. A medial–caudal–ventral approach will be used, progressively treating deeper toward the lamina of the caudal vertebrae while monitoring for reproduction of symptoms (local or referred pain or sensation) and for a local twitch response (LTR). A LTR is characterized by a contraction of part of the taut band when needling a sensitive area within a TrP and is often used clinically to confirm the needle has reached a trigger point [58,59]. Since deeper muscles in the posterior cervical region are inaccessible to palpation, the LTR will be used as an objective sign of the TrP location. For the suboccipital muscles, only the obliquus capitis inferior will be needled as it is the only one that can be safely needled due to the proximity of the vertebral artery above the posterior arch of the atlas and the foramen magnum. With the patient in the prone position, the needle will be inserted inferiorly and laterally to the target TrP between the transverse process of C1 and the spinous process of C2, in the medial half of the muscle towards the posterior arch of C1 using a cranial-medial technique to direct the needle towards the patient’s opposite eye (Figure 2) [60,61].

**Figure 3:**
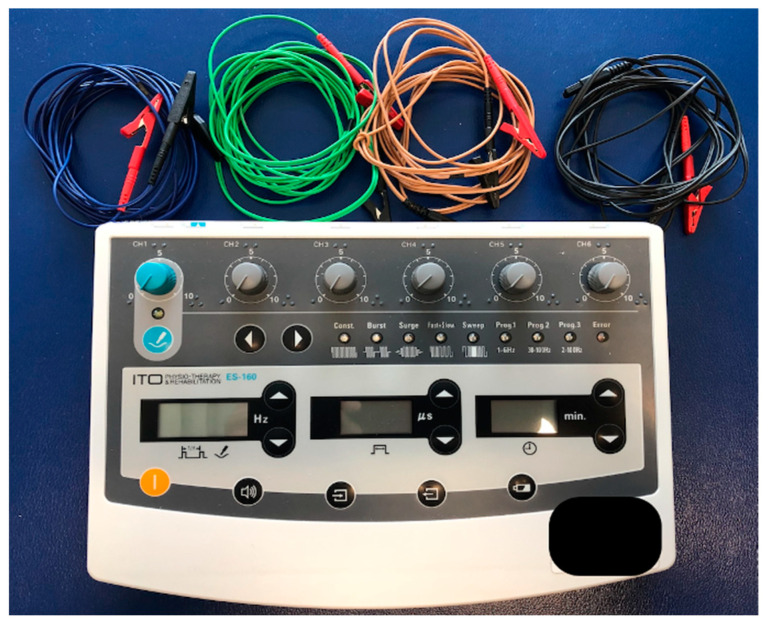
ITO ES-160 unit.

Following elicitation of an LTR, needles will be left in place and connected to a charge generating device, the ITO ES-160 unit (manufactured by ITO Co., Ltd., Tokyo, Japan), Figure 3. The parameters for IMES will be alternating frequencies of 6 Hz and 20 Hz using a symmetrical biphasic waveform (also known as a dense-and-disperse mode), which will be used in place of fixed frequencies. Several studies have reported a more robust analgesic effect using mixed frequencies in place of single (fixed) frequencies [62,63,64]. An intensity will be chosen that will elicit a “strong but not painful” response, usually within the range of 0.5 to 6 mA [65,66] with pulse durations of 300 µs, as longer pulse durations are more likely to activate high threshold small diameter afferent nerves by overcoming factors related to nerve membrane resistance [67]. In addition, longer pulse durations have been shown to produce more robust analgesic effects [68]. A treatment duration of fifteen minutes will be selected for IMES to TrPs, as durations of 15–30 min are suggested to be more effective than durations < 15 min [69,70].

### 2.6. Outcome Measures

#### 2.6.1. Primary Outcome Measure

For this study, the reduction in headache frequency, which is the reduction in the number of headache days over a one-month period, will be the primary outcome measure [71]. The primary end point for this study is the 6th, and final, visit at the end of the intervention phase where the change in the headache frequency will be assessed. Headache diary reports will be sent to the principal investigator each week during the baseline and intervention periods of the study. The headache diary reports will be sent through a secure electronic patient portal as a PDF report generated through the Migraine Buddy smartphone application. Use of this application for research purposes has increased in recent years to capture headache data and for outcome monitoring [72,73]. Headache characteristics, triggers, and medication use will also be recorded in the headache diary with a time stamp feature for data collection in real time. All headache diary data will be analyzed and stored on a Microsoft Excel spreadsheet, with all participant information de-identified. A migraine day will be defined as a day with a headache that lasts at least 4 h, which meets the International Classification of Headache Disorders 3rd edition criteria for migraine without aura, migraine with aura, and probable migraine, or a day with a headache that is successfully treated with migraine-specific acute medication [36].

#### 2.6.2. Secondary Outcome Measures

Secondary outcome measures will include the Headache Impact Test-6 (HIT-6) and the Migraine Disability Assessment Test (MIDAS). The HIT-6 is a six-item questionnaire assessing headache severity and change in a participant’s clinical status over a short period of time (4 weeks) [74]. The MIDAS is a self-administered questionnaire designed to quantify headache-related disability over a 3-month period [75]. Changes in PPTs to TrPs that correlate with migraine headache symptoms will also be analyzed [43,76]. In addition, changes in cervical musculoskeletal impairments with AROM, and performance on the CCFT will be measured. Lastly, the reduction in average headache pain intensity over a 1-month period will be assessed using the Numerical Pain Rating Scale (NPRS). The NPRS is used to rate the intensity of the headache pain. It is a scale featuring 11-points, with 0 indicating no pain and ten indicating pain as intense as one could imagine. The NPRS has been validated for use in measuring migraine severity [77]. The mean headache intensity will be calculated by dividing the sum of all NPRS scores on headache days and the mean of all noted headache days.

### 2.7. Data Analysis

#### 2.7.1. Visual Analysis

In this SCED study, the primary and secondary outcome measures will be analyzed separately using both visual and statistical analyses. First, we will conduct an exploratory data analysis to observe within- and between-phase patterns in the data with emphasis on level (mean score for the data), trend (slope), variability (spread of data points), and overlap (calculation of points overlapping between phases) [29,31,78]. The stability of data points will be assessed following the completion of the baseline phase [78]. Individual participant data will be represented using data visualization methods, including a graphical plot for each outcome variable both within and across participants. Non-overlap indices will be used to further assess overlap and will include non-overlap of all pairs (NAP) or the baseline-corrected Tau-U, depending on the baseline trend [79]. In addition, NAP, or baseline-corrected Tau-U will be used to measure effect size [31,80].

#### 2.7.2. Statistical Analysis

A longitudinal mixed-effects statistical model (also referred to as hierarchical or multilevel modeling) will be used to analyze the overlap between baseline to post-intervention for all outcome measures [81], provided that the data allows for such an analysis. A mixed-effects model can account for individual differences through the inclusion of random effects and is known to have increased statistical power by its ability to use all available data points.

### 2.8. Blinding

Neither the participants nor the principal investigator will be blinded to baseline period allocation. Due to the nature of the intervention, the clinician (principal investigator) will not be blinded to the intervention. Statistical analyses will be conducted by an independent statistician.

### 2.9. Adverse Events and Monitoring

Monitoring for adverse events (AEs) will be performed at each treatment visit by the principal investigator and will be recorded on the participants’ behalf using the headache diary. Participants will be given an informative handout describing what steps to take in the event of an adverse reaction to dry needling, and participants will be advised to immediately seek medical attention and notify the principal investigator and their physician. Possible minor side effects of dry needling may include temporary pain during needling, numbness or tingling, minor bleeding, bruising, drowsiness, tiredness, dizziness, and possible fainting [82]. More significant adverse events include pneumothorax and cervical epidural hematoma [83,84]. However, none of the muscles selected for treatment in this study are in proximity to the lung field. If a subject demonstrates an increase in the average headache pain intensity of more than two points on the NPRS following the first week of treatment or if a subject reports an increased number of consecutive headache days above their reported weekly average, the principal investigator will consult the patient and recommend discontinuation of the study.

## 3. Discussion

### 3.1. Study Implications

In this exploratory study, our primary aim is to determine if a five-week intervention with IMES to TrPs in the posterior cervical muscles of participants with chronic migraine leads to a reduction in the headache frequency. Our secondary aims are to assess the effects of IMES on headache pain intensity, migraine disability, overall headache impact, and on selected cervical musculoskeletal impairments, including PPTs, CCFT, and cervical AROM. Trigger points may serve as a perpetuating factor of migraine symptoms by activating nociceptive pathways that project to convergent dorsal horn neurons in the TCC. On the other hand, in some cases, TrP-related pain could merely be comorbid with chronic migraine and represent a nociplastic phenomenon that is more reflective of central sensitization [85]. Based on our review of the literature, IMES may exert its therapeutic effects to TrPs by normalizing the muscle blood flow, decreasing the endplate noise, and improving the function of the descending pain modulatory system [27]. Aside from the therapeutic effects elicited within TrPs, IMES may also stimulate peripheral nerves near the suboccipital and posterior cervical muscles, which may trigger segmental inhibitory effects. In addition, IMES has been shown to engage descending pain inhibitory systems that project to second order neurons in the TCC, by activating the ventrolateral division of the periaqueductal grey [86]. For example, the obliquus captitis inferior muscle is anatomically related to the greater occipital nerve [87]. We hypothesize that IMES will serve to reduce the nociceptive impact on the TCC from TrPs in the posterior cervical muscles and promote a reduction in migraine symptoms. To our knowledge, no multiple-baseline SCED study has investigated the effects of IMES to TrPs in the posterior cervical muscles in patients with chronic migraine.

### 3.2. Limitations

This study design has several limitations. First, participants will be enrolled in a non-concurrent manner due to the impracticality of having all patients begin the baseline measures simultaneously. Second, as we have stated previously, neither the participants nor the principal investor will be blinded in any phase of this study. Absent or ineffective blinding in dry needling studies has the potential to exaggerate the specific effectiveness of dry needling interventions [88,89]. Assessor blinding is one conceivable way to reduce bias and is recommended to enhance SCED quality [31]. Alas, a recent systematic review of SCED studies reported that participant/therapist blinding and blinding of assessors is a prevalent limitation in SCED studies [30]. Third, no short- or long-term follow-up was factored into this study, due to the limited time and resources needed for a longer study duration. Fourth, because each participant will be exposed to the same dry needling and IMES parameters, we will be unable to make comparisons between the effects of most parameters. Only variations in the posterior cervical muscles harboring TrPs and the electrical stimulation intensity required to elicit appropriate responses may be present between participants. Fifth, we are unable to determine the additive effect that the electrical stimulation will have on dry needling, as all participants will receive both dry needling and IMES. Lastly, although the equipment we have selected for measurements of cervical AROM have been found reliable for clinical use, only the cervical range of motion device (CROM) has shown excellent intra-rater and inter-rater reliability in patients with migraine for measures of cervical AROM and for use with the FRT [90]. Due to lack of resources available we were unable to approve the purchase of this device.

### 3.3. Potential Contributions

Current management for chronic migraine relies heavily on prophylactic drug treatments, yet the adherence to preventative medications in patients with chronic migraine is low and worsens with the duration of medication use [91]. Since there is some evidence for dry needling to TrPs and for the use of electrical stimulation in the conservative treatment of migraine, combining dry needling with electrical stimulation in this study may provide insights of their impact on headache frequency, without relying on an increased dosage of medication. A potential benefit to society is to gain a new understanding about the benefits of more conservative migraine treatments. Findings from this study may also benefit healthcare professionals who treat or oversee patients with migraine, by providing knowledge of an intervention that can be administered for selected patients within this headache population. In addition, this research may promote further investigation on IMES to TrPs in patients with migraine in larger sample sizes of participants with randomized controlled trials.

## 4. Conclusions

This study will seek to determine the effects of IMES to TrPs in the posterior cervical muscles of participants with chronic migraine. Intramuscular electrical stimulation over a five-week intervention period could demonstrate an improvement in headache outcomes in patients with chronic migraine.

## Data Availability

Data sharing is not applicable to this article.
